# 

**Published:** 1859-10

**Authors:** 


### RECEIPTS FOR THE JOURNAL, UP TO OCT. 6, 1859.

| | | | |
|---|---|---|---|
| **Ill.** | —Dr. D. E. Foot, | v. 2, '59, | $2 |
| | " L. N. Dimmock, | " | 2 |
| | " V. G. Stevens, | " | 2 |
| | " P. A. Allaire, | " | 2 |
| | " J. Welch, | " | 2 |
| | " A. A. Barrows, | " | 2 |
| | " Max Myers, | " | 2 |
| | " T. B. Trower, | " | 2 |
| | " E. C. Ellett, | " | 2 |
| | " P. A. Rosenberger, | " | 2 |
| | " J. B. Samuel, | " | 2 |
| | " J. Van Dyke, | " | 2 |
| | " Moore and Kennedy, | " | 2 |
| | " E. Richards, | v. 2–3, '59–'60, | 4 |
| | " G. W. Phillips, | v. 2 & ½3, '59–'60, | 2 |
| | " B. J. Dunn, | " " " | 2 |
| | " Wm. Law, | v. 1–2, '58–'59, | 4 |
| **Ill.** | —Dr. K. E. Rich, | v. 1, '58, | $2 |
| | " J. W. Leffingwell, | v. 1–2, '58–'59, | 4 |
| | " E. Wenger, | v. 1–3, 58–'60, | 5 |
| | " G. S. Barrows, | v. 1–2, '58–'59, | 4 |
| | " B. Woodward, | balance 2, '59, | 1 |
| **Iowa.** | —Dr. John Low, | v. 2, '59, | $2 |
| | " Ireland, | " | 2 |
| | " J. Doron, | " | 2 |
| **Ind.** | —Dr. J. H. Newland, | " | 2 |
| | " C. M. Richmond, balance, | " | 1 |
| | " John Lewis, | " | 2 |
| | " D. Hutchinson, | " | 2 |
| | " W. H. Sullivan, | 2 & ½3 '59–'60, | 2 |
| | " J. & D. E. Evans, | 2–3, '59–'60, | 4 |
| **Ark.** | —Dr. L. C. White, | v. 1–2, '58–'59, | 4 |
| **Wis.** | —Dr. L. B. W. Johnson, | v. 1–2, '58–'59 | 4 |
| | " M. Waterhouse, | balance 2, '59, | 1 |

DIED,

On Saturday, Aug. 20th, at Amboy, Lee county, Ill., HARMON WASSON, M.D., graduate of Rush Medical College, of the class of 1849.

